# Complete blood count parameters as biomarkers of retinopathy of prematurity: a Portuguese multicenter study

**DOI:** 10.1007/s00417-023-06072-7

**Published:** 2023-05-02

**Authors:** Mariza Fevereiro-Martins, Ana Carolina Santos, Carlos Marques-Neves, Hercília Guimarães, Manuel Bicho, GenE-ROP Study Group, Conceição Afonso, Joana Ferreira, Rita Espírito Santo, Filipa Teixeira, Rita Rosa, Cristina Vaz Carneiro, Marta Ferreira, Teresa Matos, Luísa Neiva, Sandra Pereira, Sofia Aires, Ricardo Parreira, Zuzana Melnik, João Faria, Joana Teixeira, Pedro Barros, Juliana Almeida, Bruna Malheiro, Patrícia Cunha Rodrigues, Luís Albuquerque, Alice Freitas, Pedro Barros, Nadezda Kotchekova, Rui Freitas, Ana Cristina Silveira, Ana Ferreira, Benvinda Morais, Susana Teixeira, Mafalda Mota, Maria Guerra, Lúcia Coimbra, João Gigante, Muriel Ferreira, Patrícia Lapa, Madalena Monteiro, Mário Alfaiate, Teresa Rodrigues, Teresa Pina, Marta Rosário, Renato Silva, Jorge Breda, Filipa Bazenga, João António Pinto

**Affiliations:** 1 Laboratório de Genética and Grupo Ecogenética e Saúde Humana, Instituto de Saúde Ambiental (ISAMB), Faculdade de Medicina, Universidade de Lisboa, Av. Professor Egas Moniz, Piso 1C, 1649-028 Lisbon, Portugal; 2 Instituto de Investigação Científica Bento da Rocha Cabral, Calçada Bento da Rocha Cabral 14, 1250-012 Lisbon, Portugal; 3 Departamento de Oftalmologia, Hospital Cuf Descobertas, Rua Mário Botas, 1998-018 Lisbon, Portugal; 4 Centro de Estudos das Ciências da Visão, Faculdade de Medicina, Universidade de Lisboa, Av. Professor Egas Moniz, Piso 1C, 1649-028 Lisbon, Portugal; 5 Grupo Ecogenética e Saúde Humana, Instituto de Saúde Ambiental (ISAMB), Faculdade de Medicina, Universidade de Lisboa, Av. Professor Egas Moniz, Piso 1C, 1649-028 Lisbon, Portugal; 6 Departamento de Ginecologia - Obstetrícia e Pediatria, Faculdade de Medicina, Universidade do Porto, Alameda Prof. Hernâni Monteiro, 4200-319 Porto, Portugal

**Keywords:** Retinopathy of prematurity, Complete blood count, Biomarker, Preterm infant

## Abstract

**Purpose:**

To evaluate complete blood count (CBC) parameters in the first week of life as predictive biomarkers for the development of retinopathy of prematurity (ROP).

**Methods:**

Multicenter, prospective, observational study of a cohort of preterm infants born with gestational age (GA) < 32 weeks or birth weight < 1500 g in eight Portuguese neonatal intensive care units. All demographic, clinical, and laboratory data from the first week of life were collected. Univariate logistic regression was used to assess risk factors for ROP and then multivariate regression was performed.

**Results:**

A total of 455 infants were included in the study. The median GA was 29.6 weeks, and the median birth weight was 1295 g. One hundred and seventy-two infants (37.8%) developed ROP. Median values of erythrocytes (*p* < 0.001), hemoglobin (*p* < 0.001), hematocrit (*p* < 0.001), mean corpuscular hemoglobin concentration (*p* < 0.001), lymphocytes (*p* = 0.035), and platelets (*p* = 0.003) of the group of infants diagnosed with ROP any stage were lower than those without ROP. Mean corpuscular volume (MCV) (*p* = 0.044), red blood cell distribution width (RDW) (*p* < 0.001), erythroblasts (*p* < 0.001), neutrophils (*p* = 0.030), neutrophils-lymphocytes ratio (*p* = 0.028), and basophils (*p* = 0.003) were higher in the ROP group. Higher values of MCV, erythroblasts, and basophils remained significantly associated with ROP after multivariate regression.

**Conclusion:**

In our cohort, the increase in erythroblasts, MCV, and basophils in the first week of life was significantly and independently associated with the development of ROP. These CBC parameters may be early predictive biomarkers for ROP.


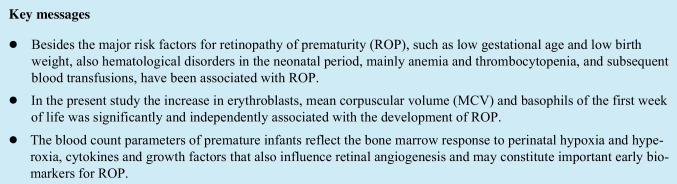


## Introduction

Retinopathy of prematurity (ROP) is a retinal vasoproliferative disease affecting preterm infants, especially those born with low birth weight [1]. Despite progressive improvements in perinatal care and therapeutic approaches, ROP remains a leading cause of potentially preventable visual impairment and blindness in children worldwide [2]. Blencowe et al. estimated that 184,700 preterm infants developed any stage of ROP in 2010 worldwide, of which 32,000 became blind or mildly/moderately visually impaired due to ROP [3]. Of the latter, 65% were born in middle-income regions [3].

ROP is recognized as a biphasic disease that results from an interruption of the retinal neurovascular development due to preterm birth [4]. In the postnatal period, partial pressure levels of oxygen are higher compared to in utero levels [5]. Suppression of vascular endothelial growth factor (VEGF) due to relative hyperoxia and the loss of maternal–fetal interaction leads to an arrest of retinal vascularization (phase 1) [6]. The generation of reactive oxygen species can cause apoptosis of vascular endothelial cells leading to vaso-obliteration [7]. Subsequently, the increasingly metabolically active retina becomes hypoxic, stimulating vasoproliferation induced by VEGF and other pro-angiogenic growth factors (phase 2), which can lead to retinal detachment [8].

ROP screening guidelines are primarily based on birth weight (BW) and gestational age (GA). However, many studies have suggested other risk factors, including maternal, prenatal, perinatal, genetic, nutritional, demographic, and others related to medical interventions and comorbidities of prematurity [4, 9–14].

Currently, the diagnosis of ROP and the decision on the need for treatment depend mainly on ophthalmologists with extensive experience in this area, resulting in some subjectivity and variability [15]. The primary screening method for ROP is based on binocular indirect ophthalmoscopy. The wide-field digital retinal imaging system is an alternative method for preliminary screening of ROP or as an adjunct to binocular indirect ophthalmoscopy. An accurate, simple, and reliable diagnostic method for diagnosing ROP has not been found [16]. The development of molecular diagnostic techniques that early identify preterm infants at risk of developing ROP will allow the timely implementation of preventive and therapeutic measures for ROP and a more individualized approach. It would also enable unnecessary evaluation of premature infants to be avoided and would save human resources [17].

In recent years, some parameters obtained from the complete blood count (CBC) of preterm infants have been investigated to find biomarkers of the development of ROP [18–25]. These parameters were generally measured at different time points, and early biomarkers predictive of ROP development have not yet been identified [25].

This study aimed to evaluate the correlation between CBC parameters in the first week of life and the development of ROP in a population of preterm infants.

## Methods

### Population

This is a multicenter, prospective, observational, and analytical study based on a Portuguese cohort from 8 neonatal intensive care units (Centro Hospitalar Universitário de Lisboa Norte, Hospital Prof. Doutor Fernando Fonseca, Centro Hospitalar Universitário de São João, Centro Materno Infantil do Norte belonging to the Centro Hospitalar Universitário do Porto, Hospital de Braga, Hospital da Senhora da Oliveira—Guimarães, Maternidade Bissaya Barreto, and Maternidade Daniel de Matos belonging to the Centro Hospitalar Universitário de Coimbra). This is a registered observational study (ISRCTN16889608).

The recruitment period started in a phased manner in the various hospital centers, between 19 November 2018 and 19 December 2019, ending between 30 December 2020 and 21 July 2021.

This study followed from birth the consecutive sampling of preterm infants regardless of sex and race, with at least one of the following characteristics: (1) born before 32 weeks of GA; (2) born with BW of less than 1500 g. The Exclusion criteria were as follows: (1) major congenital malformations in the preterm infant; (2) ophthalmological pathologies, congenital or acquired (during the first 12 weeks of life) not related to ROP except for conjunctivitis, keratitis, and congenital nasolacrimal duct obstruction; (3) death before the first ROP screening; (4) insufficient clinical data due to patient transfer to another hospital; (5) absence of informed consent from parents or legal guardians.

### ROP screening and ophthalmological data collection

The first retinal examination was performed at each neonatal intensive care unit by ophthalmologists qualified for ROP screening at 31–33 weeks of postmenstrual age (PMA) or 4 to 6 weeks after birth, whichever was later. The following methods and procedures were used: (1) Adequate mydriasis was obtained through the administration of eye drops according to the guidelines of the latest consensus on ROP of the Portuguese Society of Neonatology [26]; (2) The primary screening was performed by indirect binocular ophthalmoscopy or digital fundus retinography (RetCam) or both, depending on the indication and the hospital center; (3) Examination results were documented for each eye separately according to the criteria of the International ROP Classification (ICROP), published in 1984 [27], and revisited in 2005 [28], and were described in terms of location (zones), the severity of retinopathy (stages), the extent of lesions in the active phase, and the presence or absence of plus disease; (4) Subsequent examinations were determined in each case according to the existence, zone, and severity of ROP and were repeated until complete retinal vascularization or until complete remission of ROP after treatment. Follow-up examinations, the highest ROP stage, and any treatment required were also recorded for both eyes according to the ICROP guidelines [27–29]. The guidelines introduced by the Early Treatment for Retinopathy of Prematurity study (ETROP) [30] were followed regarding the treatment criteria. All patients diagnosed with ROP type 1 were treated with LASER photocoagulation or anti-VEGF, depending on the location of the disease in the retina and the hospital center.

### Other data

Demographic, clinical, and laboratory data were collected. CBC from the first week of life was determined according to standardized methods. CBC included erythrocytes (× 1012/L); hemoglobin (g/dL); hematocrit (%); mean corpuscular volume (fl); mean corpuscular hemoglobin concentration (pg); red blood cell distribution width (%); erythroblasts (× 10^9^/L); leukocytes(× 10^9^/L); neutrophils (%); lymphocytes (%); neutrophils-lymphocytes ratio; basophiles (%); eosinophils (%); platelets (× 10^9^/L); and plateletcrit (%). Data related to the objectives were collected from hospital medical records during hospitalization.

Pediatric data included the following: (1) antenatal steroids; (2) birth data: GA, gender, body weight; (3) respiratory data: duration of oxygen administration and mechanical ventilation, need for surfactant therapy; (4) presence of comorbidities such as sepsis, meningitis, bronchopulmonary dysplasia, periventricular and intraventricular hemorrhage, cystic periventricular leukomalacia, patent ductus arteriosus with hemodynamic significance, and necrotizing enterocolitis; (5) number of packed red blood cells, platelets, and serum transfusions received; (6) number of days with hyperglycemia (defined as blood glucose > 125 mg/dL in total blood or > 150 mg/dL in serum) in the first 3 weeks of life.

### Statistical analysis

All continuous variables were tested for normality using the Kolmogorov–Smirnov test. Since all were non-normal, median and interquartile range were presented for these tests. In categorical groups, the Mann–Whitney test was used for comparisons between groups. Logistic regression analysis and the corresponding 95% CI were calculated using a binary dependent variable to model the probability of a risk factor for no ROP or ROP or no ROP vs. stages 1 and 2 ROP vs. stage 3 ROP vs. type 1 ROP.

Univariate logistic regression was used to evaluate the risk factors for ROP. The risk factors considered were GA, BW, sex, preeclampsia/eclampsia, gestational diabetes, hyperglycemia, hyaline membrane disease, days of oxygen ventilation, postnatal steroids, red blood cell transfusion, days of red blood cell transfusions, platelet transfusions, days of platelet transfusions, bronchopulmonary dysplasia, persistent ductus arteriosus, periventricular and intraventricular hemorrhage, sepsis, and necrotizing enterocolitis.

After, a multivariate regression (backward conditional) was performed using variables that were significant in univariate and clinically meaningful. Statistical analysis was performed with the SPSS program with a significant value of *p* < 0.05.

### Ethics approval

The study protocol, data collection form, and informed consent were approved by the Scientific Council of the Faculty of Medicine of the University of Lisbon and by all scientific councils of participating hospital centers, and by the Ethics Committee of Centro Hospitalar Universitário de Lisboa Norte and Centro Académico de Medicina de Lisboa (CAML) (reference number 340/2018), and Ethics Committees of all other participating hospital centers (Ethics Committee of Hospital da Senhora da Oliveira Guimarães reference number 01/19-CAc, Ethics Committee of Centro Hospitalar Universitário de São João reference number 20–19, Ethics Committee of Hospital Professor Doutor Fernando Fonseca reference number 70/2018, Ethics Committee of Hospital de Braga reference number 15/2019, Ethics Committee of Centro Hospitalar e Universitário de Coimbra reference number CHUC-014–019, Ethics Committee of Centro Hospitalar Universitário do Porto reference number 031-DEFI/032-CE). All collected data is stored and treated as confidential clinical information under the anonymity of the participants. Written informed consent was obtained from the parents of all infants included in the study. The study was performed in accordance with the ethical standards as laid down in the 1964 Declaration of Helsinki and its later amendments or comparable ethical standards.

## Results

**Fig. 1:**
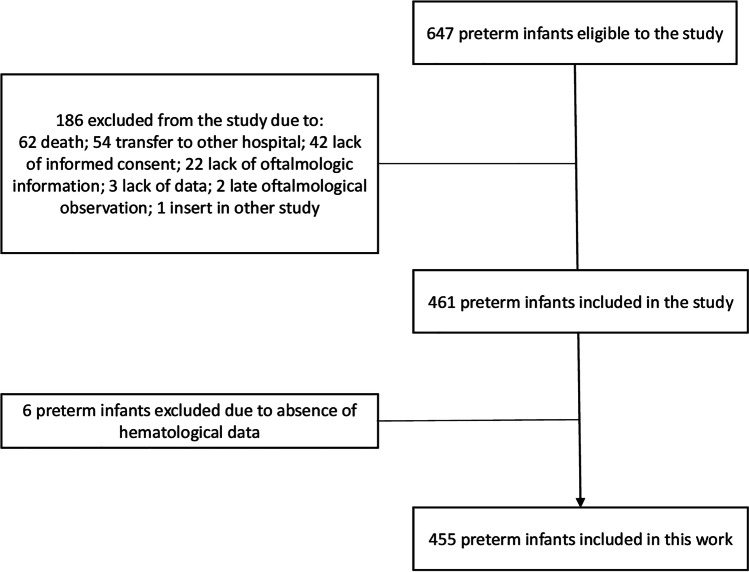
Flowchart representative of the eligibility and inclusion of preterm infants in the study

A total of 647 preterm infants were eligible for this study; however, only 461 met the criteria to be included due mainly to death or transfer to other hospitals and consequent follow-up loss (Fig. 1). In this cohort, 455 preterm infants were included, with 230 (50.5%) females. In our study, the incidence of ROP was 37.7%, and no preterm infant developed stage 4 or 5 ROP. A total of 283 (62.3%) preterm infants did not had ROP, stages 1 and 2 represented 133 (29.2%), and stage 3 and type 1 ROP were observed in 18 (3.9%) and 21 (4.6%) preterm infants, respectively (Table 1).

**Table 1:** Demographic and clinical characteristics stratified by stages of ROP

Clinical characteristics, *n* (%) or median (interquartile range)	Total	No ROP	Stage 1 and stage 2 ROP	Stage 3 ROP	Type 1 ROP	*p* value
Number of individuals, *n* (%)	455 (100%)	283 (62.3)	133 (29.2)	18 (3.9)	21 (4.6)	
Gestational age (weeks)	29.6, 3.2	30.4, 2.4	28.4, 3.0	26.4, 2.3	26.1, 2.1	**< 0.001**
Birth weight (g)	1175.7,445.0	1295.0, 370.0	1050.0, 423.5	850.0, 560.0	755.0, 283.5	**< 0.001**
Gender						0.506
Male	225 (49.5)	147 (51.9)	59 (44.4)	8 (44.4)	11 (52.4)	
Female	230 (50.5)	136 (48.1)	74 (55.6)	10 (55.6)	10 (47.6)	
Bronchopulmonary dysplasia	92 (20.2)	28 (9.9)	39 (29.3)	8 (44.4)	17 (81.0)	**< 0.001**
Necrotizing enterocolitis	26 (5.7)	11 (3.9)	8 (6.0)	6 (33.3)	1 (4.8)	**< 0.001**
Days with glycemia ≥ 125 mg/dL	1.0, 3.0	2.0, 3.0	3.0, 5.0	5.0, 6.0	5.0, 4.0	**< 0.001**
Antenatal steroids	416 (91.4)	261 (92.2)	119 (89.5)	16 (88.9)	20 (95.2)	0.802
Pulmonary surfactant	219 (48.1)	92 (32.5)	95 (71.4)	13 (72.2)	19 (90.5)	** < 0.001**
Oxygen ventilation	402 (88.9)	234 (82.7)	129 (97.0)	18 (100.0)	21 (100.0)	**0.001**
Days of oxygen ventilation	24.0, 46.0	9.0, 31.0	37.0, 44.0	71.0, 53.0	91.0, 46.0	**< 0.001**
Red blood cell transfusions	164 (36.0)	50 (17.7)	79 (59.4)	14 (77.8)	20 (95.2)	**< 0.001**
Days of red blood cell transfusions	2.0, 3.0	1.0, 1.0	2.0, 9.0	4.0, 3.0	5.0, 4.0	**< 0.001**
Platelets transfusions	43 (9.3)	7 (2.5)	26 (19.5)	2 (11.1)	8 (38.1)	**< 0.001**
Days of platelets transfusions	1.0, 1.0	2.0, 1.0	1.0, 1.0	2.0, NA	1.5, 1.0	**< 0.001**
Plasma transfusions	15 (3.3)	2 (0.7)	9 (6.8)	3 (16.7)	1 (4.8)	**< 0.001**
Days of plasma transfusions	1.0, 1.0	1.5, NA	1.0, 0.0	2.0, NA	1.0, 0.0	**< 0.001**

*n* number of individuals; *p* value, no ROP vs stage 1 + 2 ROP vs stage 3 ROP vs type 1 ROP. *NA* not applicable

*p*-value less than 0.05 are in bold

The median of the cohort for the GA was 29.6 weeks and for the BW was 1175.7 g. The median values of GA and BW decreased as the severity of ROP increased (Table 1).

Table 1 includes the clinical characteristics, where it was observed that 416 (91.4%) of the mothers used antenatal steroids, and 219 (48.1%) of preterm infants needed pulmonary surfactant. The red blood cell and platelet transfusion frequency were higher in type 1 ROP, while the plasma transfusions were more frequent in stage 3 ROP. Regarding cell transfusions, red blood cells were more frequent than platelets and plasma.

Regarding prematurity-related diseases, bronchopulmonary dysplasia affected 92 (20.2%) preterm infants of the study population. The need for oxygen ventilation was observed in 402 (88.9%) of the preterm infants, and the number of days of oxygen ventilation was higher in the stage 3 and type 1 ROP (Table 1).

Table 2 shows the association between parameters of CBC in preterm infants without or with ROP. Various parameters of the CBC were associated with the development of ROP.

**Table 2:** Association of parameters of complete blood count with absence or presence of ROP

Parameters of complete blood count (median, interquartile range)	ROP	No ROP	*p* value	*p* value^#^	*p* value*	*p* value¤
Number of individuals, *n* (%)	172 (37.8)	283 (52.2)	NA	NA	NA	NA
Gestational age (weeks)	28.0, 2.9	30.4, 2.4	**< 0.001**	NA	NA	**< 0.001**
Birth weight (g)	975.0, 410.0	1295.0, 370	**< 0.001**	**0.039**	**0.002**	**< 0.001**
Erythrocytes (× 10^12^/L)	4.0, 0.8	4.3, 0.8	**< 0.001**	0.096	0.502	0.600
Hemoglobin (g/dL)	14.8, 3.5	16.1, 3.4	**< 0.001**	0.215	0.941	0.881
Hematocrit (%)	43.7, 9.8	45.5, 9.1	**0.001**	**0.027**	0.207	0.335
Mean corpuscular volume (fl)	108.2, 11.4	107.7, 9.7	**0.044**	**0.023**	**0.034**	**0.017**
Mean corpuscular hemoglobin concentration (pg)	37.2, 4.2	37.8, 3.5	**< 0.001**	0.685	** < 0.001**	** < 0.001**
Red blood cell distribution width (%)	17.0, 2.9	16.5, 2.2	**0.001**	**0.038**	**0.001**	0.117
Erythroblasts (× 10^9^/L)	14.9, 30.6	8.0, 16.7	**< 0.001**	**0.037**	**0.010**	**0.026**
Leukocytes (× 10^9^/L)	9.3 (7.0)	9.2 (4.8)	0.599	0.817	0.790	0.225
Neutrophils (%)	42.6, 17.9	41.1, 15.2	**0.030**	0.681	0.697	0.707
Lymphocytes (%)	37.0, 20.1	40.3, 17.3	**0.035**	0.902	0.990	0.388
Neutrophils-lymphocytes ratio	1.1, 1.2	0.9, 0.8	**0.028**	0.682	0.361	0.504
Basophiles (%)	0.7, 0.7	0.5, 0.4	**0.003**	**0.005**	**0.012**	**0.004**
Eosinophils (%)	1.8 (2.3)	2.1 (2.5)	0.132	0.120	0.143	0.116
Platelets (× 10^9^/L)	201.7, 118.0	222.7, 104.7	**0.003**	**0.044**	**0.005**	0.054
Plateletcrit (%)	0.2, 0.1	0.2, 0.1	0.001	0.919	0.864	0.891

*n* number of individuals; *p* value^#^—adjusted for gestational age and days of red blood cell transfusions. *NA* not applicable; *p* value*—adjusted for gestational age; *p* value¤—adjusted for days of red blood cell transfusions

*p*-value less than 0.05 are in bold

Regarding erythrocyte parameters, the increase in mean corpuscular volume (MCV) and erythroblasts was significantly associated with the group that later developed ROP, even after adjusting for gestational age and number of days of red blood cell transfusion, risk factors determined by the multivariate regression.

Other parameters related to the erythrocyte lineage, such as the decrease in erythrocytes, hemoglobin, hematocrit, and MCHC, were shown to be associated with the development of ROP; however, they were statistically dependent on gestational age and/or days of red blood cell transfusions. Likewise, higher red blood cell distribution width (RDW) was significantly associated with developing ROP but dependent on days of red blood cell transfusions.

On the other hand, increased basophils were significantly associated with developing ROP in logistic regression analysis.

Neutrophilia, lymphopenia, and increased neutrophil-to-lymphocyte ratio were associated with the development of ROP, but the result was not statistically significant in regression analysis.

Thrombocytopenia was a risk factor for developing ROP, but dependent on days of red blood cell transfusions.

## Discussion

In the present study, the incidence of ROP at any stage was 37.7%. The incidence of ROP in studies is variable, even comparing countries with similar neonatal intensive care levels. This is due to the great variability in the study designs, GA of preterm infants included, survival rates, and treatments used [8]. Two other prospective studies with similar inclusion criteria, one in the USA and another in Brazil, had an incidence of ROP of 36.6% (211/576) and 25.9% (91/352), respectively [31, 32]. In another recent Portuguese study, the incidence of ROP was 23.8% [33], which may be because it was a retrospective study (between 2012 and 2020) and involved only two hospital centers.

Low GA and low BW were significant risk factors for the development of ROP (Table 1) in agreement with the results of several other studies [1], confirming the value of these parameters as the main determinants of ROP*.* Our results also showed that hemoderivative transfusion, BPD, oxygen therapy, surfactant, and number of days with hyperglycemia are significantly associated with the risk of developing ROP and its progression. The same results were presented in other studies [5, 34–36].

Concerning erythrocyte parameters, we observed that the increase in MCV and erythroblasts was significantly and independently associated with the group that later developed ROP (Table 2).

A significant relationship between most fetal hematological values and GA is described. MCV normally decreases while hematocrit increases with GA [37]. Although in our study the group that developed ROP had a lower mean GA than the group that did not develop ROP, it was observed that the association between the increase in MCV and ROP was statistically significant after adjustment in the logistic regression analysis.

To our knowledge, this is the first study reporting a relationship between increased erythroblasts and the development of ROP. However, an increase in both erythrocyte precursors, such as erythroblasts and reticulocytes, and nucleated erythrocytes generally reflects increased erythropoietic activity. Lubetsky et al. and Niranjan et al. observed that the increase in the nucleated red blood cell count on the first day of life can be used to predict ROP and that it can be associated with intrauterine hypoxia [18, 38]. According to these investigators, this result is presumably due to an increased compensatory erythropoiesis as a consequence of intrauterine hypoxia. Erythropoietin regulates the production of fetal erythrocytes in order to maintain normal erythropoiesis at steady state [39]. Serial measurements of erythropoietin levels in the amniotic fluid have shown that when the fetus becomes hypoxic, the concentration of erythropoietin in the amniotic fluid begins to increase exponentially [39].

Other parameters, such as erythrocytes, hemoglobin, hematocrit, and MCHC, were associated with the development of ROP, but were statistically dependent on gestational age and/or days of red blood cell transfusion. In the study by Ünsal et al., the referred analytical parameters evaluated in the fourth week of life were significantly associated with the development of ROP [40]. In the study by Kurtul et al., there was no association between hemoglobin and hematocrit on the first day of life and ROP [19]. Although, in relation to this last study, fluctuations in blood volume and hematocrit on the first day of life make it difficult to interpret the hematocrit value. In the first several postnatal hours, there is a transudation of plasma volume, reducing blood volume and increasing venous hematocrit, after which blood volume increases [41].

In our study, we also found RDW significantly associated with ROP development but dependent on days of red blood cell transfusions, another important risk factor for the development of ROP [5]. RDW corresponds to the coefficient of variation of red blood cell volume, reflecting the heterogeneity of erythrocyte volume, and this may explain why the association we found between RDW and ROP is modulated by days of red blood cell transfusions [24].

Other studies showed that increased RDW was associated with the development of ROP. The increase in RDW in the first 2 weeks of life in the study by Çömez et al. and in the fourth week of life in the study by Ünsal et al. was significantly associated with the development of ROP [24, 40]. In the study by Said et al. carried out with children admitted to the Saint Louis Children’s Hospital Pediatric Intensive Care Unit, RDW was associated with greater morbidity and mortality [42]. In the course of critical illness, red blood cells acquire metabolic and structural lesions, which can lead to decreased deformability, increased adhesion to the endothelium, increased affinity for oxygen [42, 43], decreased hemoglobin content [42, 44], decreased antioxidant capacity [42, 45], restriction of energy metabolism, and impairment of nitric oxide processing and its role in vascular signaling [42, 46]. For this reason, RDW has been suggested as a possible biomarker for RBC damage of sufficient magnitude to influence critical illness outcome, likely by affecting oxygen supply [42]. The increase in RDW, as it can be a biomarker of the disturbance in the supply of oxygen to tissues, and in particular to the retina, may be predictive for the development of ROP [24].

To the best of our knowledge, we describe for the first time a relationship between basophilia and ROP. The increase in basophils was significantly and independently associated with the development of ROP. Basophils constitute about 1% of human peripheral blood leukocytes and have a short half-life [47]. Like other myeloid lineages, they are produced from hematopoietic stem cells in the bone marrow, circulate in peripheral blood, and are rarely present in tissues unless inflammation occurs [47].

Human basophils when activated by immunologic stimuli produce a restricted profile of cytokines such as IL-4 [48], IL-13 [49], and IL-3 [50] and also pro-angiogenic molecules such as VEGF-A, VEGF-B, angiopoietin 1 (ANGPT1), C-X-C motif chemokine ligand 8 (CXCL8), and hepatocyte growth factor [47]. It is also known that human basophils express on their surface VEGF receptor 2 (VEGFR-2) and the co-receptors neuropilin 1 and neuropilin 2 [51]. These data suggest that angiogenesis can be modulated by human basophils [51, 52]. According to the findings of our study, it is possible that the activation of basophils in the context of inflammation due to factors related to prematurity may play a role in the development of ROP.

In this study, thrombocytopenia in the first week of life was shown to be a risk factor for the development of ROP but dependent on days of red blood cell transfusions. It is presumed that, due to red blood cell transfusions, replacement of fetal hemoglobin by adult hemoglobin with lower affinity for oxygen may result in a higher fraction of dissolved oxygen in plasma and greater exposure of tissues to oxygen [53]. This may downregulate the production of erythropoietin, an angiogenic and oxygen-regulated growth factor that promotes erythropoiesis and plays a role in thrombopoiesis. Furthermore, increased oxygen delivery to the retina is an important risk factor for ROP [5, 54].

Parrozani et al. observed an association between thrombocytopenia in the first hour of life in preterm infants and type 1 ROP [25]. An association between thrombocytopenia and type 1 ROP was also observed from the first day of life to 36 weeks of postmenstrual age in the study by Cakir et al. and 1 week before laser in the study by Jensen et al. [55, 56]. In the study by Akdogan et al., thrombocytopenia at 1 month, but not on the first day of life, was associated with the development of ROP [23].

Despite recent studies linking thrombocytopenia to ROP, the role of platelets in ROP is still not fully understood. Platelets contain several alpha granules with pro-angiogenic molecules, such as VEGF and IGF-1, and anti-angiogenic molecules, such as endostatin [25, 56]. This compartmentalization of different angiogenic regulators within platelets, which can be selectively released or sequestered in different local environments, may explain how platelets can stimulate or inhibit angiogenesis [25, 56]. Moreover, platelet count is highly correlated with serum levels of VEGF-A and brain-derived neurotrophic factor (BDNF), supporting the hypothesis that platelet count could be used as an indirect measure of platelet-derived levels of these factors [57].

In the first week of life of preterm infants, when we analyzed the platelet count, the retina was still avascular and needed to be more vascularized. It is possible that at this stage platelets may have a more stimulating role in angiogenesis by releasing IGF-1, and that thrombocytopenia may cause an imbalance between the mediators of angiogenesis. Hellgren et al. showed that in preterm infants, maintaining platelet levels and, therefore, platelet-derived growth factors can reduce the risk of developing ROP [57].

Our results suggest that some CBC parameters, beyond their traditional clinical applicability, may be early predictive biomarkers of ROP. Confirmation of these results may allow the integration of these biomarkers in models to calculate the risk of developing ROP. The use of biomarkers and possibly also clinical risk factors in a risk analysis system could potentially allow the development of more appropriate screening and follow-up strategies for preterm infants, depending on their risk level. It could also reduce the amount of ROP screening tests for lower-risk infants, sparing them this discomfort while also saving human resources.

As limitations of this study, it should be noted that, due to its observational nature, CBCs were obtained from routine tests performed on preterm infants or from clinical indications unrelated to the study, in the first week of life, but without defined time points for their performance. Furthermore, as this is a multicenter study, the screening for ROP was performed by experienced ophthalmologists, but different for each hospital center. As such, the existence of bias due to inter-observer variability cannot be excluded.

More prospective studies with larger cohorts are needed to confirm our results.

In conclusion, we found some parameters of the CBC of the first week of life as possible major early predictive biomarkers of the development of ROP. In particular, the increase in erythroblasts, MCV, and basophils was significantly and independently associated with the development of ROP. These results are encouraging regarding the use of CBC parameters as a widely available and cost-effective screening test that could potentially be integrated into models to provide preliminary guidance to ophthalmologists and neonatologists about infants at risk of developing ROP, which is now more necessary than ever.

## Abbreviations

BW: Birth weight
CBC: Complete blood count
GA: Gestational age
MCV: Mean corpuscular volume
RDW: Red blood cell distribution width
ROP: Retinopathy of prematurity
VEGF: Vascular endothelial growth factor
